# Normative Data on Cognitive Performance in Urban and Rural Populations of Kazakhstan: A Cross-Sectional Analysis

**DOI:** 10.1007/s10823-026-09608-6

**Published:** 2026-11-05

**Authors:** Adil Supiyev, Ángela Gutiérrez, Zhaxybay Zhumadilov, Graciela Muniz-Terrera

**Affiliations:** 1 Ohio University Heritage College of Osteopathic Medicine, Medical Education Center 118, 169 W. Union St., Athens, Ohio 45701 USA; 2 Department of Health Sciences, College of Health Sciences, Western University of Health Sciences, Pomona, CA 91766 USA; 3 Nazarbayev University School of Medicine, Kabanbay Batyr Ave. 53, Astana, 010000 Kazakhstan

**Keywords:** Cognitive function, Normative data, Neuropsychological tests, Aging, Urban-rural differences, Kazakhstan

## Abstract

Objectives. Evidence on cognitive performance among older adults in Kazakhstan and Central Asia remains limited. This study aimed to establish normative values for neuropsychological tests among adults living in urban and rural areas of Kazakhstan, stratified by age and educational attainment. Methods. This cross-sectional study included 1,648 adults aged 50–75 years randomly selected from urban and rural communities in Central and East Kazakhstan between 2012 and 2016. Four standardized neuropsychological tests assessed verbal memory and learning, verbal fluency, attention, and concentration. Normative values were calculated separately by urban-rural residence, age group, and educational level. Results. Cognitive performance generally declined with increasing age and was higher among participants with greater educational attainment. Urban residents demonstrated higher mean scores than rural residents on immediate word recall (18.98 vs. 16.49), animal naming (18.17 vs. 16.37), and delayed word recall (6.32 vs. 5.71), whereas rural residents scored higher on letter cancellation (22.06 vs. 19.76; all p < 0.001). Age-, education-, and residence-specific percentile norms were established for all four cognitive tests. Discussion. This study provides some of the first population-based normative data for multiple cognitive domains among urban and rural adults in Kazakhstan. These reference values may support the interpretation of cognitive assessments in comparable Central Asian populations, although further validation and studies involving older age groups are needed.

**Supplementary Information:**

The online version contains supplementary material available at https://doi.org/10.1007/s10823-026-09608-6.

## Introduction

Cognitive decline and age-related cognitive disorders, such as Alzheimer’s disease and related dementias (ADRD), are significant concerns among the older population in Central Asia. Like many other developing regions, Central Asian countries are witnessing a demographic transition as a result of increasing life expectancy and improving healthcare (Liou, Joe et al. 2020). With a larger segment of the population reaching older ages, there is a need to improve the understanding of cognitive functioning of these older individuals and identify those who are impaired or at increased risk of cognitive impairment, to address the specific needs and challenges they face.

Normative cognitive scores can provide valuable insights into an individual’s cognitive abilities by comparing their performance to established norms (Giulioli, Meillon et al. 2016, Kahali, Balakrishnan et al. 2023). However, the use of local normative data of a specific population is recommended for more meaningful interpretation of the results (Casaletto, Umlauf et al. 2015). Normative data for cognitive function specific to Kazakhstan and other Central Asian countries is scarce (Casaletto, Umlauf et al. 2015, Kulimbet, Glushkova et al. 2022) or has been produced either from small samples or is limited due to their focus only on urban populations. In addition, normative data may not be readily available for many cognitive assessment tools, especially given the specific cultural and linguistic context of post-soviet countries.

Kazakhstan is the ninth-largest country in the world by land area (Zhumadilov 2012) and provides a distinctive historical, geographical, and socioeconomic context for examining cognitive performance among older adults. Individuals in the age groups examined in this study spent much of their formative years under Soviet governance and subsequently experienced substantial social and economic transformations following Kazakhstan’s independence in 1991 (Zhumadilova, Zhakupova et al. 2026). These historical circumstances may have shaped educational opportunities, living conditions, and other life-course experiences relevant to cognitive performance, particularly across urban and rural populations. This context further highlights the importance of developing population-specific cognitive normative data that account for age, educational attainment, and urban-rural residence.

Therefore, the aim of the present study was to derive normative scores for cognitive tests among middle-aged and older adults living in urban and rural communities in Kazakhstan, stratified by age group, education level, and place of residence. We used data collected between 2012 and 2016 as part of a population-based health study conducted in Central and East Kazakhstan. The study included participants from urban and rural communities in both regions and collected information on cognitive and physical functioning, anthropometric measurements, socioeconomic characteristics, lifestyle factors, and other health-related indicators. By providing normative cognitive scores across different demographic and residential groups, this analysis aims to contribute to the interpretation of cognitive performance in populations for which locally derived reference values remain limited.

## Methods

### Study Population

We used data from a population-based health study conducted between 2012 and 2016 in Central and East Kazakhstan. The study included two urban-rural pairs: Astana city and Akmol village in Central Kazakhstan, and Semey city and Borodulikha village in East Kazakhstan. The Astana-Akmol population has been described in previous population-based investigations (Supiyev, Kossumov et al. 2016, Zhumadilova, Zhakupova et al. 2026), while the present analysis additionally includes participants from East Kazakhstan.

The data were collected as part of a broader research project on health and aging in Kazakhstan that originated as the Astana Health Study and subsequently expanded into the Kazakhstan Longitudinal Aging Study (KazLAS). The original project was established in late 2012 in Astana city and the surrounding Akmola region, with participant recruitment beginning in 2013 and most baseline examinations conducted in 2014–2015. The project included two complementary components: a population-based cross-sectional study of adults aged 50–75 years from urban and rural communities and a case-control study involving consecutively recruited patients with acute coronary syndrome and stroke, with controls drawn from the population-based sample. The project subsequently expanded its geographical coverage to East Kazakhstan, with additional population-based assessments conducted in Semey city and Borodulikha village during 2015–2016. Longitudinal follow-up of the original Astana-Akmol population-based cohort was initiated in 2024, approximately ten years after the main baseline examinations, and remains ongoing. Previous publications have described the original Astana-Akmol population and selected cardiometabolic outcomes (Supiyev, Kossumov et al. 2015, Supiyev, Kossumov et al. 2016, Supiyev, Nurgozhin et al. 2017). The present analysis derives cognitive normative values exclusively from cross-sectional assessments of the population-based sample, without including clinical cases or longitudinal follow-up measurements.

The study sample was randomly selected, after stratification by sex and five-year age groups, from residents aged 50–75 years registered at local polyclinics. Participants were invited to participate by telephone or, when no landline telephone number was available, through home visits. A total of 1,648 adults were included in the present analysis: 486 from Astana city, 467 from Akmol village, 397 from Semey city, and 298 from Borodulikha village. The previously published Astana-Akmol study reported an overall response rate of approximately 59% (Supiyev, Kossumov et al. 2016).

Data were collected during visits to local polyclinics, where participants completed standardized questionnaires administered by trained practitioners and underwent physical and cognitive assessments. The questionnaires included information on general health, medical history, lifestyle, and socioeconomic characteristics. All questionnaire items were translated from English into Russian and Kazakh and back-translated into English to check for accuracy. Cognitive function was assessed using four standardized neuropsychological tests, and physical function was assessed using three tests. Blood pressure and anthropometric measurements, including height, weight, waist circumference, and hip circumference, were obtained. Venous blood samples were also collected. The study protocol was approved by the Ethics Committee of the Center for Life Sciences at Nazarbayev University, Kazakhstan, and all participants provided informed consent.

### Cognitive Function

The protocol for cognitive assessments was adopted from the HAPIEE study (Health, Alcohol and Psychosocial factors In Eastern Europe), one of the largest prospective studies of non-communicable diseases in Eastern Europe, with a major focus on healthy aging and relevant study instruments applicable to other post-soviet countries (Peasey, Bobak et al. 2006, Horvat, Richards et al. 2014).

*Cognitive function* was assessed by trained practitioners using four neuropsychological tests (Table 1):

**Table 1:** Cognitive tests used to derive normative scores

Test	Cognitive domain	Variable used	Abbr.	Score range
Immediate recall (10-word list recall, 3 consecutive tests)	Verbal memory and learning	Total correct answers	IWR	0–30
Animal naming	Verbal fluency	Total correct answers	AN	0-max
Letter cancellation	Attention, mental speed and concentration	Total correct answers	LC	0–65
Delayed recall (10-word list recall)	Verbal memory and learning	Total correct answers	DWR	0–10

1. Verbal memory and learning were assessed using a 10-word list recall. Word lists, consisting of 10 common nouns, were the same in each recruiting site in Astana and Semey regions. Immediate word recall was assessed by counting the correctly recalled words summed over 3 consecutive 1-minute trials (range 0-30).
2. Verbal fluency was assessed by asking participants to name as many animals as possible within 1 minute. Names of different species counted toward the total but redundancies (e.g., black cow, brown cow) did not. Total number of correct responses was recorded.
3. Attention, mental speed, and concentration: Letter cancellation test was used to assess these cognitive functions. Participants were instructed to cross out 2 target letters, “P” and “Ш” in Cyrillic, embedded in a random letter A4 grid as quickly and as accurately as possible within 1 minute. Total number of letters correctly crossed out was used (range 0-65).
4. Delayed recall: a10-word delayed list recall was assessed after an interval during which previous cognitive tests were administered, recall was assessed by correctly recalled words (range 0-10).

The cognitive assessment instruments were adopted from the Russian-language version of the HAPIEE study protocol, translated into Kazakh, and piloted before implementation to evaluate linguistic clarity and cultural appropriateness. Both language versions were offered to participants, who selected their preferred language for assessment. Russian was more commonly selected, likely reflecting the historical and educational background of this older population, many of whom were educated during the Soviet period when Russian was widely used. To ensure consistency in test administration, members of the research team visited the study sites to provide in-person training to local interviewers and directly conducted some of the assessments.

### Data Analysis

The sociodemographic characteristics and cognitive test scores of participants were summarized using descriptive statistics, including frequencies, percentages, means, and standard deviations. Participants were categorized into five age groups (50–54, 55–59, 60–64, 65–69, and 70–75 years) and three educational levels: university (Higher), vocational/secondary (Apprenticeship), and primary education or less (Primary). Urban residence included Astana and Semey cities, while rural residence included Akmol and Borodulikha villages. Differences in sociodemographic characteristics and cognitive test scores between urban and rural participants were assessed using independent-samples t-tests for continuous variables and Pearson’s chi-square tests for categorical variables. Welch’s t-test was used where unequal variances were assumed. Multiple linear regression analyses were performed to examine the associations of age (as a continuous variable), sex, education (as an ordinal numerical variable), and urban-rural residence with neuropsychological test scores. Following previous literature (Giulioli, Meillon et al. 2016), normative scores were derived separately for urban and rural participants, stratified by age group and educational level. The 5th, 10th, 25th, 50th, and 75th percentiles were calculated for each cognitive test. Participants with missing cognitive test scores were excluded from the corresponding test-specific analyses. All statistical analyses and visualizations were performed using Stata software, version 18 (StataCorp, College Station, TX, USA).

## Results

Normative cognitive scores were calculated using data from 1,648 participants recruited from urban and rural locations in two geographical regions of Kazakhstan (Central Kazakhstan-Astana region and East Kazakhstan-Semey region). Table 2 presents the sociodemographic characteristics and cognitive test scores of the study population. The mean age was 61.26 years (SD = 7.51), and approximately 60% of participants were female. Urban residents comprised 53.6% of the sample and were slightly older than rural residents (61.77 [SD = 7.82] vs. 60.68 [SD = 7.10] years; *p* = 0.003). Most participants were recruited from the Astana region (57.8%) and were slightly younger than participants from the Semey region (60.88 [SD = 7.17] vs. 61.78 [SD = 7.94] years; *p* = 0.017). Overall, 27.9% of participants had completed higher education, 38.7% had vocational/secondary education, and 33.4% had primary education or less. Participants aged 50–54 years represented the largest age group (25.8%), whereas those aged 70–75 years comprised the smallest proportion (15.7%).

**Table 2:** Sociodemographic characteristics and cognitive performance by urban-rural residence (*N* = 1,648)

Characteristic	Total	Urban	Rural	*P*-value
Participants, n	1,648	883	765	
Age, mean (SD)	61.26 (7.51)	61.77 (7.82)	60.68 (7.10)	0.003
Sex, n (%)				< 0.001
Male	660 (40.05)	394 (44.62)	266 (34.77)	
Female	988 (59.95)	489 (55.38)	499 (65.23)	
Education, n (%)				< 0.001
Higher	459 (27.85)	347 (39.30)	112 (14.64)	
Apprenticeship	638 (38.71)	325 (36.81)	313 (40.92)	
Primary	551 (33.43)	211 (23.90)	340 (44.44)	
Age groups, n (%)				0.001
50–54	425 (25.79)	226 (25.59)	199 (26.01)	
55–59	356 (21.60)	163 (18.46)	193 (25.23)	
60–64	320 (19.42)	168 (19.03)	152 (19.87)	
65–69	289 (17.54)	164 (18.57)	125 (16.34)	
70–75	258 (15.66)	162 (18.35)	96 (12.55)	
Cognitive tests, mean (SD)				
Immediate word recall	17.78 (4.30)	18.98 (3.92)	16.49 (4.32)	< 0.001
Animal naming	17.29 (5.49)	18.17 (5.61)	16.37 (5.21)	< 0.001
Letter cancellation	20.90 (8.68)	19.76 (7.00)	22.06 (9.99)	< 0.001
Delayed word recall	6.02 (1.92)	6.32 (1.90)	5.71 (1.90)	< 0.001

Values are n (%) or mean (SD). Percentages are within residence groups. P-values compare urban and rural participants using independent-samples t-tests or Pearson chi-square tests; Welch’s t-test was used for letter cancellation. Cognitive test sample sizes: immediate word recall *n* = 1,539; animal naming *n* = 1,531; letter cancellation *n* = 1,221; delayed word recall *n* = 1,542

Sociodemographic characteristics differed significantly between urban and rural participants, including age (*p* = 0.003), sex (*p* < 0.001), education (*p* < 0.001), and age-group distribution (*p* = 0.001). Urban participants demonstrated higher mean scores for immediate word recall (18.98 vs. 16.49), animal naming (18.17 vs. 16.37), and delayed word recall (6.32 vs. 5.71) compared with rural participants (all *p* < 0.001). In contrast, rural participants had higher mean scores on the letter cancellation test (22.06 vs. 19.76; *p* < 0.001). These findings indicate differences in cognitive performance across urban and rural settings, although the direction of these differences varied by cognitive domain.

Table 3 presents the results of multiple linear regression analyses examining the associations of age, sex, education, and urban-rural residence with neuropsychological test scores. Increasing age was generally associated with poorer cognitive performance across the assessed domains. Lower educational attainment was also associated with poorer cognitive performance. Associations with sex varied across cognitive tests, while urban residence was generally associated with higher cognitive scores, except for letter cancellation, for which the association was in the opposite direction.

**Table 3:** Multiple regression models for neuropsychological test scores

	IWR	AN	LC	DWR
Age	-0.14(-0.16, -0.11)**	-0.14(-0.18, -0.11)**	-0.37(-0.43, -0.31)**	-0.05(-0.07, -0.04)**
Sex	1.04 (0.65, 1.42)**	-0.35 (-0.86, 0.17)	1.09 (0.18, 1.99)*	0.55 (0.37, 0.74)**
Education level	-1.17 (-1.42, -0.92)**	-1.74 (-2.08, -1.41)**	-1.81 (-2.40, -1.22)**	-0.43 (-0.55, -0.31)**
Residence	-2.18 (-2.57, -1.78)**	-1.149 (-1.68, -0.62)**	2.84 (1.90–3.78)**	-0.53 (-0.72, -0.35)**

Values are expressed as unstandardized regression coefficients (β) with 95% confidence intervals. *IWR* Immediate word recall, *AN*  Animal naming, *LC*  Letter cancellation, *DWR*  Delayed word recall

Age was analysed as a continuous variable. Sex was categorized as male or female, with male as the reference category. Education was classified into three ordered categories (higher, apprenticeship, and primary education) and entered as an ordinal numerical variable in the regression models. Residence was categorized as urban or rural, with urban as the reference category

*p ≤ 0.05; **p ≤ 0.001

**Fig. 1:**
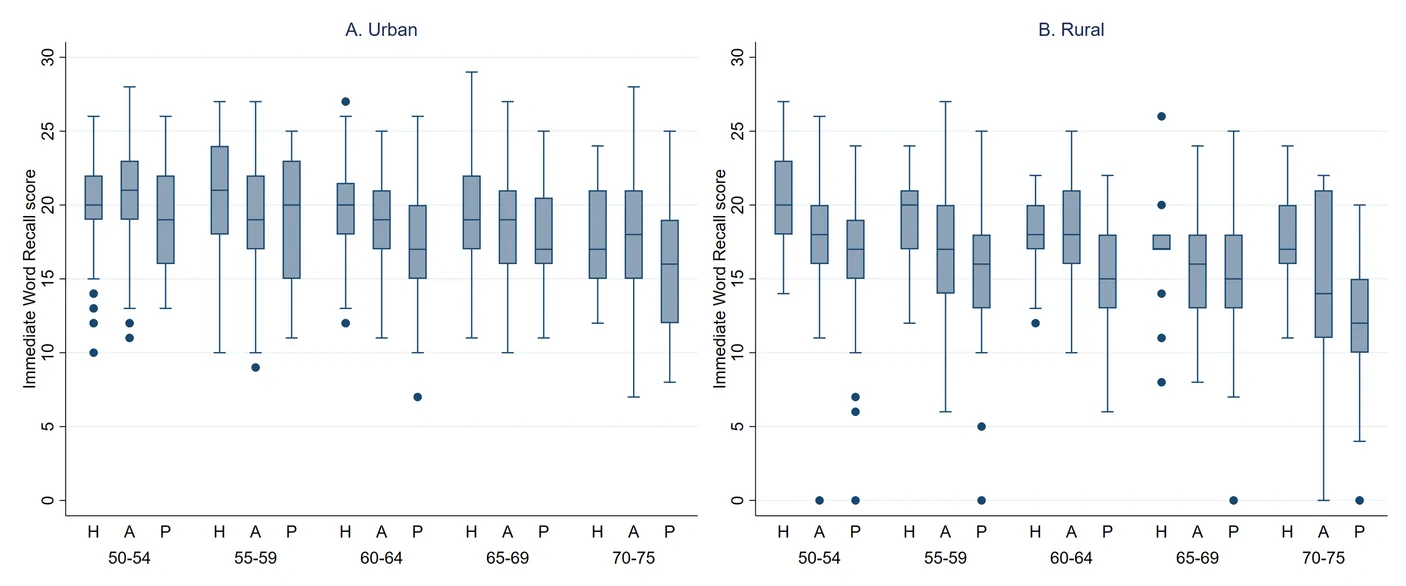
Distribution of immediate word recall scores by age group and educational attainment among urban (**a**) and rural (**b**) participants. H = Higher education; A = Apprenticeship (vocational/secondary education); P = Primary education or less. Boxes represent the interquartile range, horizontal lines within boxes indicate the median, whiskers extend to the most extreme observations within 1.5 times the interquartile range, and points indicate outliers

**Fig. 2:**
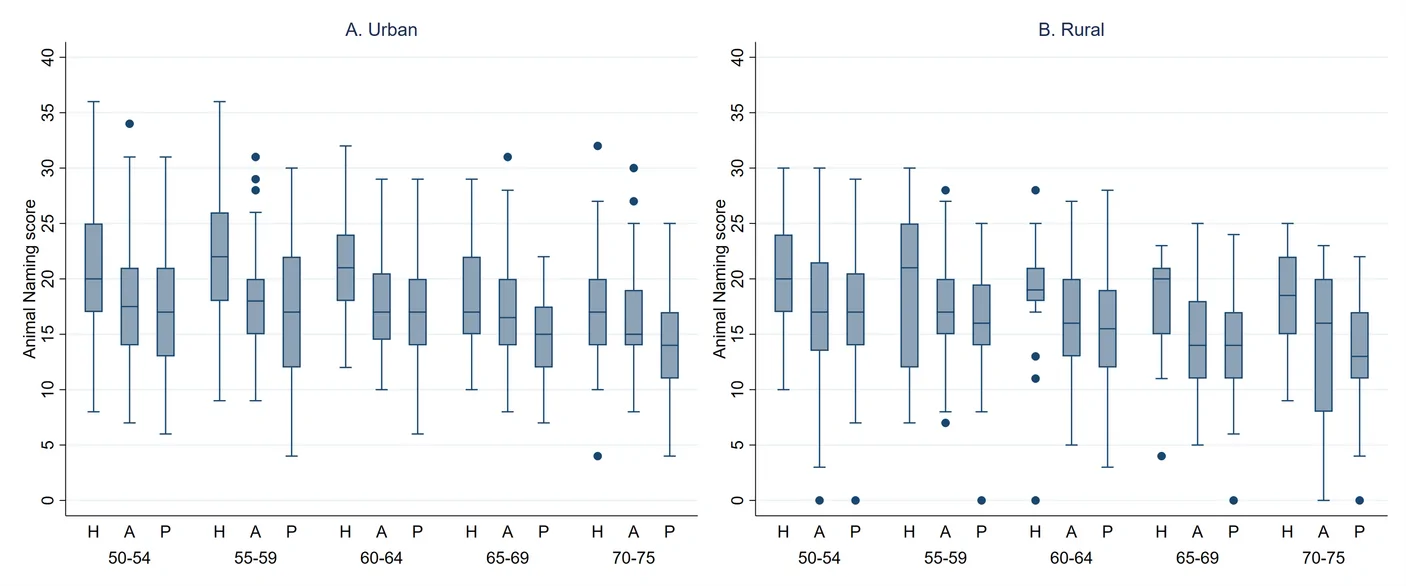
Distribution of animal naming scores by age group and educational attainment among urban (**a**) and rural (**b**) participants. H = Higher education; A = Apprenticeship (vocational/secondary education); P = Primary education or less. Boxes represent the interquartile range, horizontal lines within boxes indicate the median, whiskers extend to the most extreme observations within 1.5 times the interquartile range, and points indicate outliers

**Fig. 3:**
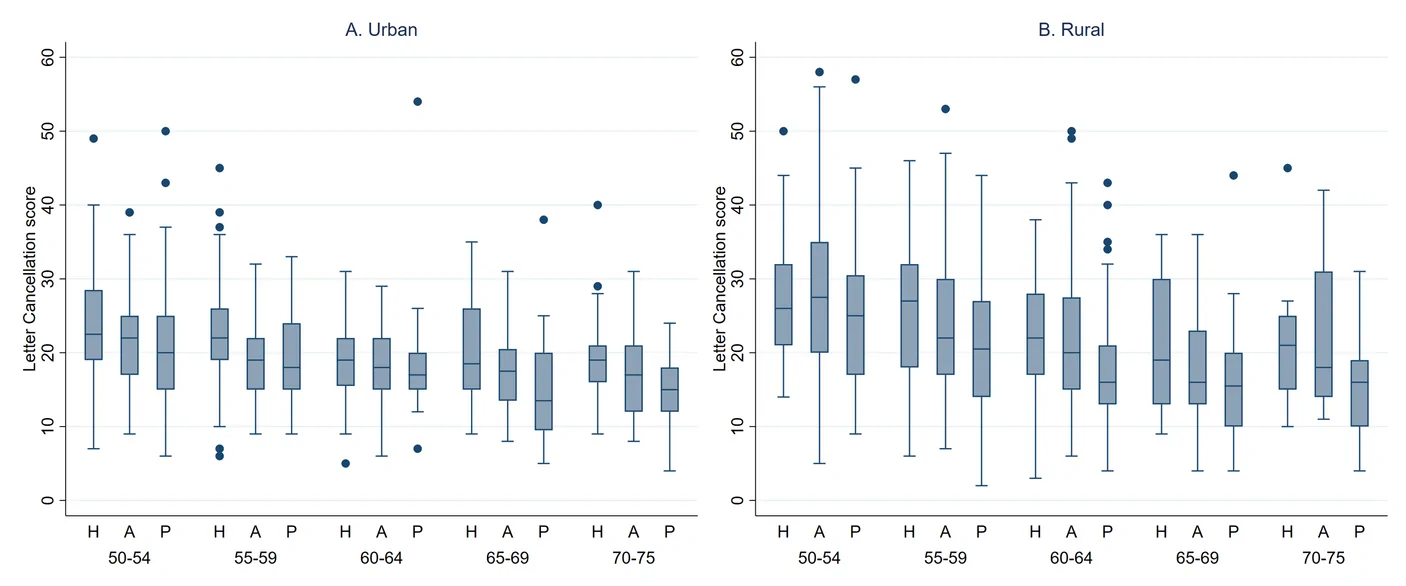
Distribution of letter cancellation scores by age group and educational attainment among urban (**a**) and rural (**b**) participants. H = Higher education; A = Apprenticeship (vocational/secondary education); P = Primary education or less. Boxes represent the interquartile range, horizontal lines within boxes indicate the median, whiskers extend to the most extreme observations within 1.5 times the interquartile range, and points indicate outliers

**Fig. 4:**
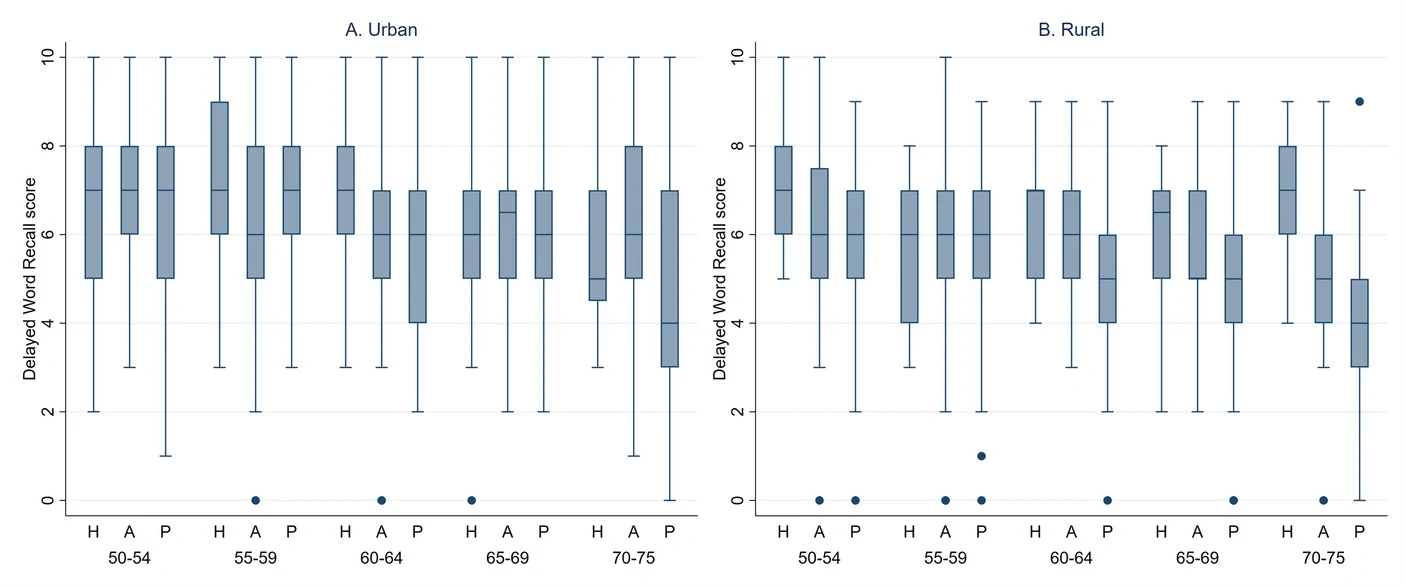
Distribution of delayed word recall scores by age group and educational attainment among urban (**a**) and rural (**b**) participants. H = Higher education; A = Apprenticeship (vocational/secondary education); P = Primary education or less. Boxes represent the interquartile range, horizontal lines within boxes indicate the median, whiskers extend to the most extreme observations within 1.5 times the interquartile range, and points indicate outliers

Normative scores for each cognitive test are presented separately for urban and rural participants, stratified by five age groups (50–54, 55–59, 60–64, 65–69, and 70–75 years) and three educational levels (Higher, Apprenticeship, and Primary) in Supplementary Tables S1 and S2, respectively. The number of participants included in each cognitive test varied because some participants were unable to complete certain assessments due to visual or hearing difficulties, difficulties understanding the tasks, or refusal to participate. The distributions of cognitive test scores, including medians, interquartile ranges, minimum and maximum values, and outliers, are illustrated by residence, age group, and educational attainment in Figs. 1a-b, 2a-b and 3a-b, and 4a-b. The 5th percentile represents the score at or below which approximately 5% of participants performed, whereas the 75th percentile represents the score at or below which approximately 75% of participants performed.

Overall, cognitive performance was generally highest among participants with higher education, lowest among those with primary education or less, and intermediate among those with vocational/secondary education. Cognitive test scores generally declined with increasing age. Differences between urban and rural participants were also observed, with urban participants performing better on immediate word recall, animal naming, and delayed word recall, while rural participants obtained higher scores on letter cancellation.

## Discussion

In the present study, we present normative data for four neuropsychological tests among 1,648 adults aged 50–75 years living in urban and rural settings in Central and East Kazakhstan. To our knowledge, this is among the first large population-based studies to establish cognitive performance norms for older adults in Kazakhstan, stratified by age, educational attainment, and urban-rural residence. The cognitive assessment protocol was adapted from the HAPIEE study and administered in Russian or Kazakh according to participants’ language preferences, taking into account the linguistic and cultural context of Kazakhstan and other post-Soviet countries. Cognitive performance was generally higher among participants with higher educational attainment and lower among older age groups. Differences were also observed between urban and rural participants, with urban residents obtaining higher scores on immediate word recall, animal naming, and delayed word recall, while rural residents performed better on the letter cancellation test (Supplementary Tables S1 and S2; Figs. 1a-b, 2a-b and 3a-b, and 4a-b).

Differences in cognitive performance between urban and rural residents were particularly evident in verbal memory and learning and verbal fluency, whereas the direction of the difference was reversed for letter cancellation, which assesses attention, mental speed, and concentration. Urban participants were slightly older and more highly educated than rural participants, with approximately 39% of urban residents having completed higher education compared with 15% of rural residents. Similar urban-rural differences in cognitive performance have been reported in previous studies (Saenz, Downer et al. 2018).These differences may partly reflect variations in educational opportunities, socioeconomic conditions, occupational characteristics, and other factors associated with urban and rural environments (Cetty, Peh et al. 2020). Astana, as the capital of Kazakhstan, has experienced substantial population growth and economic development, attracting individuals with higher educational attainment and employment in public administration and the private sector. However, the observed differences should be interpreted cautiously, as this cross-sectional analysis cannot establish causal relationships between residence and cognitive performance.

The present study has several strengths. First, it includes a relatively large population-based sample from both urban and rural communities in two geographical regions of Kazakhstan. Previous studies in the region have generally included smaller samples, focused primarily on urban populations, or assessed global cognitive function rather than individual cognitive domains (Tsoy, Turuspekova et al. 2019, Kulimbet, Glushkova et al. 2022). The inclusion of participants from both Central and East Kazakhstan provides broader geographical coverage than studies restricted to a single location. Second, the cognitive assessment protocol was adopted from the HAPIEE study, a large epidemiological investigation in Eastern Europe, and included standardized tests covering verbal memory, verbal fluency, attention, and concentration. The assessments were available in Russian and Kazakh, and the translated instruments were piloted to assess clarity and cultural appropriateness. Standardized administration procedures and training of study personnel were used to improve consistency across study locations. Third, the study included participants with a range of educational backgrounds and common cardiometabolic conditions, allowing the development of normative values that reflect variation within the study population. The broader study was also designed to support future longitudinal investigations.

Several limitations should be considered when interpreting the findings. First, the previously published Astana-Akmol study reported a response rate of approximately 59% (Supiyev, Kossumov et al. 2016). Although some individuals may not have received an invitation to participate, nonresponse and potential selection bias cannot be excluded. While response rates of this magnitude have been reported in other population-based studies (Johnson and Wislar 2012), potential selection bias cannot be excluded, particularly if nonparticipants differed systematically from participants in characteristics associated with cognitive performance. Previous population-based research has suggested that nonparticipants may have poorer health and less favorable socioeconomic characteristics than participants, potentially leading to an overestimation of cognitive performance in the study sample (Fry, Littlejohns et al. 2017). However, the extent and direction of any such selection bias could not be determined in the present study. Second, the number of participants in the older age groups was relatively small, and the study was restricted to adults aged 50–75 years. Consequently, the normative values may be less precise for older participants and cannot be directly generalized to individuals older than 75 years. The age distribution may partly reflect demographic characteristics and life expectancy patterns in the study regions; nevertheless, additional data from older age groups would improve the applicability of these norms. Third, although the cognitive instruments were translated and piloted for use in Russian and Kazakh, a comprehensive psychometric validation of the adapted instruments was not conducted. Potential differences related to language, cultural background, and educational experience may therefore influence test performance. Fourth, the number of completed assessments varied across cognitive tests because of difficulties related to vision, hearing, task comprehension, or refusal to complete particular tests. Such missing data may have affected the estimated normative distributions, particularly for tests with fewer observations. Finally, although the study included two geographical regions and both urban and rural communities, the findings may not fully represent the broader population of Kazakhstan or other Central Asian countries. Additional population-based investigations and longitudinal follow-up studies are needed to extend these normative data and evaluate changes in cognitive performance over time.

## Conclusions

This study provides age-, education-, and residence-specific normative values for four neuropsychological tests among adults aged 50–75 years in Central and East Kazakhstan. Cognitive performance generally declined with increasing age and was higher among participants with greater educational attainment. Urban residents demonstrated higher performance on verbal memory and verbal fluency tests, whereas rural residents obtained higher scores on the letter cancellation test. These findings highlight the importance of considering demographic and geographical characteristics when interpreting cognitive test results. The normative values may serve as useful reference data for cognitive assessment in comparable populations in Kazakhstan and contribute to the development of population-specific cognitive norms in Central Asia. Further research, including studies involving older age groups, additional geographical regions, and longitudinal follow-up, is needed to improve the generalizability of these reference values and better understand factors associated with cognitive performance.

## Supplementary Material

Below is the link to the electronic supplementary material.

Supplementary Material 1

## Data Availability

The data supporting the findings of this study are available from the corresponding author upon reasonable request, subject to applicable ethical and institutional restrictions.
