# Supplementary material for: Normative Data on Cognitive Performance in Urban and Rural Populations of Kazakhstan: A Cross-Sectional Analysis
Source: J Cross Cult Gerontol. 2026 Nov 5;41(4):59. doi: 10.1007/s10823-026-09608-6 (PMC13740090; doi:10.1007/s10823-026-09608-6)
Supplement: Supplementary file 1 [file 10823_2026_9608_MOESM1_ESM.docx]

**Supplementary Table S1. Age- and education-stratified normative scores for cognitive performance among urban adults in Kazakhstan.**

| **URBAN** | | | | | | | | | | | | | |
| --- | --- | --- | --- | --- | --- | --- | --- | --- | --- | --- | --- | --- | --- |
|  |  | **Immediate word recall**  **(number of correct responses)** | | | **Animal naming**  **(number of correct responses)** | | | **Letter cancellation**  **(number of correct responses)** | | | **Delayed word recall**  **(number of correct responses)** | | |
| **Age Range (years)** |  | *Higher* | *Apprentice* | *Primary* | *Higher* | *Apprentice* | *Primary* | *Higher* | *Apprentice* | *Primary* | *Higher* | *Apprentice* | *Primary* |
| **Total N** |  | *310* | *294* | *194* | *305* | *283* | *196* | *255* | *211* | *151* | *311* | *292* | *196* |
| **50-54** | **N** | **91** | **72** | **41** | **90** | **70** | **42** | **76** | **55** | **38** | **91** | **70** | **42** |
|  | **Mean** | 19.98 | 20.38 | 19.07 | 20.82 | 18.00 | 16.88 | 23.88 | 22.2 | 21.11 | 6.59 | 6.90 | 6.36 |
|  | **Std Dev** | 3.45 | 3.31 | 3.78 | 5.78 | 5.58 | 5.83 | 7.45 | 6.50 | 9.31 | 1.79 | 1.52 | 1.95 |
|  | **5** | 13 | 13 | 14 | 12 | 10 | 8 | 13 | 13 | 10 | 3 | 4 | 3 |
|  | **10** | 15 | 16 | 14 | 14 | 10.5 | 8 | 15 | 14 | 10 | 4 | 5 | 4 |
|  | **25** | 19 | 19 | 16 | 17 | 14 | 13 | 19 | 17 | 15 | 5 | 6 | 5 |
|  | **50** | 20 | 21 | 19 | 20 | 17.5 | 17 | 22.5 | 22 | 20 | 7 | 7 | 7 |
|  | **75** | 22 | 23 | 22 | 25 | 21 | 21 | 28.5 | 25 | 25 | 8 | 8 | 8 |
|  |  |  |  |  |  |  |  |  |  |  |  |  |  |
| **55-59** | **N** | **74** | **53** | **27** | **71** | **49** | **28** | **64** | **38** | **21** | **72** | **52** | **28** |
|  | **Mean** | 20.77 | 18.79 | 19.26 | 21.79 | 17.86 | 17.61 | 22.63 | 19.16 | 19.14 | 7.25 | 6.19 | 6.93 |
|  | **Std Dev** | 3.85 | 4.26 | 4.12 | 6.08 | 5.08 | 6.08 | 6.75 | 5.28 | 6.15 | 1.81 | 1.97 | 1.84 |
|  | **5** | 14 | 12 | 12 | 12 | 9 | 11 | 12 | 10 | 13 | 4 | 2 | 4 |
|  | **10** | 16 | 13 | 13 | 15 | 11 | 12 | 16 | 11 | 13 | 5 | 4 | 4 |
|  | **25** | 18 | 17 | 15 | 18 | 15 | 12 | 19 | 15 | 15 | 6 | 5 | 6 |
|  | **50** | 21 | 19 | 20 | 22 | 18 | 17 | 22 | 19 | 18 | 7 | 6 | 7 |
|  | **75** | 24 | 22 | 23 | 26 | 20 | 22 | 26 | 22 | 24 | 9 | 8 | 8 |
|  |  |  |  |  |  |  |  |  |  |  |  |  |  |
| **60-64** | **N** | **56** | **58** | **43** | **55** | **56** | **43** | **48** | **47** | **33** | **56** | **59** | **43** |
|  | **Mean** | 19.79 | 19.09 | 16.95 | 21.18 | 17.63 | 17.14 | 18.46 | 17.85 | 18.82 | 6.89 | 5.98 | 5.53 |
|  | **Std Dev** | 3.23 | 3.20 | 3.92 | 5.02 | 4.66 | 4.71 | 5.59 | 5.17 | 7.47 | 1.57 | 1.89 | 1.79 |
|  | **5** | 13 | 13 | 10 | 13 | 10 | 9 | 9 | 8 | 12 | 3 | 3 | 3 |
|  | **10** | 15 | 15 | 11 | 15 | 11 | 11 | 10 | 10 | 14 | 5 | 4 | 3 |
|  | **25** | 18 | 17 | 15 | 18 | 14.5 | 14 | 15.5 | 15 | 15 | 6 | 5 | 4 |
|  | **50** | 20 | 19 | 17 | 21 | 17 | 17 | 19 | 18 | 17 | 7 | 6 | 6 |
|  | **75** | 21.5 | 21 | 20 | 24 | 20.5 | 20 | 22 | 22 | 20 | 8 | 7 | 7 |
|  |  |  |  |  |  |  |  |  |  |  |  |  |  |
| **65-69** | **N** | **58** | **58** | **36** | **58** | **56** | **36** | **40** | **40** | **24** | **60** | **58** | **36** |
|  | **Mean** | 19.12 | 18.86 | 17.89 | 18.29 | 17.04 | 14.81 | 20.18 | 17.35 | 15.29 | 6.13 | 6.31 | 5.89 |
|  | **Std Dev** | 3.82 | 4.07 | 3.16 | 5.12 | 5.05 | 3.63 | 6.77 | 5.26 | 7.15 | 1.87 | 1.58 | 1.65 |
|  | **5** | 12 | 12 | 13 | 11 | 9 | 8 | 11 | 9.5 | 8 | 3 | 4 | 2 |
|  | **10** | 14 | 14 | 14 | 12 | 11 | 10 | 13 | 10.5 | 9 | 4 | 4 | 4 |
|  | **25** | 17 | 16 | 16 | 15 | 14 | 12 | 15 | 13.5 | 9.5 | 5 | 5 | 5 |
|  | **50** | 19 | 19 | 17 | 17 | 16.5 | 15 | 18.5 | 17.5 | 13.5 | 6 | 6.5 | 6 |
|  | **75** | 22 | 21 | 20.5 | 22 | 20 | 17.5 | 26 | 20.5 | 20 | 7 | 7 | 7 |
|  |  |  |  |  |  |  |  |  |  |  |  |  |  |
| **70-75** | **N** | **31** | **53** | **47** | **31** | **52** | **47** | **27** | **31** | **35** | **32** | **53** | **47** |
|  | **Mean** | 17.52 | 17.83 | 15.83 | 17.55 | 16.33 | 13.91 | 19.29 | 17.26 | 14.51 | 5.72 | 6.13 | 4.91 |
|  | **Std Dev** | 3.89 | 4.10 | 4.12 | 5.48 | 4.30 | 4.35 | 6.24 | 5.93 | 5.10 | 1.75 | 2.15 | 2.22 |
|  | **5** | 12 | 11 | 11 | 10 | 11 | 8 | 9 | 9 | 4 | 3 | 2 | 2 |
|  | **10** | 13 | 12 | 11 | 11 | 12 | 8 | 12 | 10 | 5 | 4 | 4 | 2 |
|  | **25** | 15 | 15 | 12 | 14 | 14 | 11 | 16 | 12 | 12 | 4.5 | 5 | 3 |
|  | **50** | 17 | 18 | 16 | 17 | 15 | 14 | 19 | 17 | 15 | 5 | 6 | 4 |
|  | **75** | 21 | 21 | 19 | 20 | 19 | 17 | 21 | 21 | 18 | 7 | 8 | 7 |

Values are presented as sample size (N), mean, standard deviation (SD), and 5th, 10th, 25th, 50th, and 75th percentiles. Normative scores are stratified by five age groups (50-54, 55-59, 60-64, 65-69, and 70-75 years) and three education levels (higher, apprenticeship/secondary, and primary or less).

**Supplementary Table 2S. Age- and education-stratified normative scores for cognitive performance among rural adults in Kazakhstan.**

| **RURAL** | | | | | | | | | | | | | |
| --- | --- | --- | --- | --- | --- | --- | --- | --- | --- | --- | --- | --- | --- |
|  |  | **Immediate word recall**  **(number of correct responses)** | | | **Animal naming**  **(number of correct responses)** | | | **Letter Cancellation**  **(number of correct responses)** | | | **Delayed word recall**  **(number of correct responses)** | | |
| **Age Range (years)** |  | *Higher* | *Apprentice* | *Primary* | *Higher* | *Apprentice* | *Primary* | *Higher* | *Apprentice* | *Primary* | *Higher* | *Apprentice* | *Primary* |
| **Total N** |  | *107* | *309* | *325* | *107* | *310* | *330* | *100* | *252* | *252* | *107* | *307* | *332* |
| **50-54** | **N** | **37** | **92** | **68** | **37** | **92** | **68** | **35** | **78** | **56** | **37** | **92** | **69** |
|  | **Mean** | 20.16 | 18.18 | 16.63 | 19.86 | 17.37 | 16.97 | 27.83 | 27.76 | 25.07 | 7.08 | 6.26 | 5.90 |
|  | **Std Dev** | 3.65 | 3.95 | 4.17 | 4.74 | 5.38 | 5.18 | 8.56 | 11.25 | 9.62 | 1.53 | 1.83 | 1.87 |
|  | **5** | 15 | 12 | 10 | 11 | 9 | 9 | 16 | 12 | 11 | 5 | 4 | 3 |
|  | **10** | 15 | 13 | 12 | 13 | 11 | 10 | 19 | 15 | 12 | 5 | 4 | 3 |
|  | **25** | 18 | 16 | 15 | 17 | 13.5 | 14 | 21 | 20 | 17 | 6 | 5 | 5 |
|  | **50** | 20 | 18 | 17 | 20 | 17 | 17 | 26 | 27.5 | 25 | 7 | 6 | 6 |
|  | **75** | 23 | 20 | 19 | 24 | 21.5 | 20.5 | 32 | 35 | 30.5 | 8 | 7.5 | 7 |
|  |  |  |  |  |  |  |  |  |  |  |  |  |  |
| **55-59** | **N** | **21** | **102** | **67** | **21** | **102** | **68** | **20** | **86** | **54** | **21** | **99** | **67** |
|  | **Mean** | 18.76 | 17.17 | 15.60 | 19.57 | 17.31 | 16.51 | 25.5 | 23.40 | 20.89 | 5.86 | 6.00 | 5.84 |
|  | **Std Dev** | 3.19 | 3.94 | 4.16 | 6.74 | 4.30 | 4.76 | 9.50 | 9.27 | 9.08 | 1.65 | 1.87 | 1.78 |
|  | **5** | 12 | 11 | 10 | 10 | 11 | 8 | 10 | 8 | 7 | 4 | 3 | 3 |
|  | **10** | 14 | 12 | 10 | 11 | 11 | 11 | 15 | 12 | 11 | 4 | 4 | 4 |
|  | **25** | 17 | 14 | 13 | 12 | 15 | 14 | 18 | 17 | 14 | 4 | 5 | 5 |
|  | **50** | 20 | 17 | 16 | 21 | 17 | 16 | 27 | 22 | 20.5 | 6 | 6 | 6 |
|  | **75** | 21 | 20 | 18 | 25 | 20 | 19.5 | 32 | 30 | 27 | 7 | 7 | 7 |
|  |  |  |  |  |  |  |  |  |  |  |  |  |  |
| **60-64** | **N** | **21** | **53** | **73** | **21** | **54** | **74** | **19** | **44** | **56** | **21** | **54** | **74** |
|  | **Mean** | 18.00 | 17.83 | 15.32 | 18.71 | 16.37 | 15.45 | 21.84 | 22.66 | 17.66 | 6.33 | 6.09 | 5.04 |
|  | **Std Dev** | 2.88 | 3.54 | 3.50 | 5.74 | 4.63 | 5.05 | 9.32 | 10.51 | 8.39 | 1.20 | 1.59 | 1.76 |
|  | **5** | 13 | 11 | 10 | 11 | 8 | 7 | 3 | 9 | 6 | 5 | 4 | 2 |
|  | **10** | 14 | 13 | 11 | 13 | 11 | 9 | 6 | 12 | 8 | 5 | 4 | 3 |
|  | **25** | 17 | 16 | 13 | 18 | 13 | 12 | 17 | 15 | 13 | 5 | 5 | 4 |
|  | **50** | 18 | 18 | 15 | 19 | 16 | 15.5 | 22 | 20 | 16 | 7 | 6 | 5 |
|  | **75** | 20 | 21 | 18 | 21 | 20 | 19 | 28 | 27.5 | 21 | 7 | 7 | 6 |
|  |  |  |  |  |  |  |  |  |  |  |  |  |  |
| **65-69** | **N** | **14** | **47** | **58** | **14** | **47** | **59** | **13** | **33** | **40** | **14** | **47** | **59** |
|  | **Mean** | 16.86 | 15.89 | 15.05 | 17.64 | 14.45 | 13.97 | 20.69 | 18.18 | 15.73 | 6.07 | 5.66 | 4.97 |
|  | **Std Dev** | 4.11 | 3.48 | 4.58 | 5.41 | 4.43 | 4.63 | 9.24 | 7.86 | 7.69 | 1.69 | 1.55 | 1.92 |
|  | **5** | 8 | 11 | 7 | 4 | 9 | 7 | 9 | 7 | 4.5 | 2 | 3 | 2 |
|  | **10** | 11 | 11 | 9 | 11 | 9 | 9 | 10 | 10 | 7 | 5 | 4 | 3 |
|  | **25** | 17 | 13 | 13 | 15 | 11 | 11 | 13 | 13 | 10 | 5 | 5 | 4 |
|  | **50** | 17 | 16 | 15 | 20 | 14 | 14 | 19 | 16 | 15.5 | 6.5 | 5 | 5 |
|  | **75** | 18 | 18 | 18 | 21 | 18 | 17 | 30 | 23 | 20 | 7 | 7 | 6 |
|  |  |  |  |  |  |  |  |  |  |  |  |  |  |
| **70-75** | **N** | **14** | **15** | **59** | **14** | **15** | **61** | **13** | **11** | **46** | **14** | **15** | **60** |
|  | **Mean** | 17.21 | 14.47 | 12.27 | 18.43 | 14.07 | 13.10 | 20.85 | 22.82 | 15.43 | 6.79 | 5.00 | 3.97 |
|  | **Std Dev** | 3.72 | 5.85 | 4.18 | 4.18 | 6.90 | 4.60 | 9.04 | 10.42 | 6.57 | 1.53 | 2.14 | 1.84 |
|  | **5** | 11 | 0 | 4 | 9 | 0 | 6 | 10 | 11 | 6 | 4 | 0 | 0.5 |
|  | **10** | 12 | 10 | 7 | 14 | 7 | 7 | 12 | 14 | 8 | 5 | 3 | 1 |
|  | **25** | 16 | 11 | 10 | 15 | 8 | 11 | 15 | 14 | 10 | 6 | 4 | 3 |
|  | **50** | 17 | 14 | 12 | 18.5 | 16 | 13 | 21 | 18 | 16 | 7 | 5 | 4 |
|  | **75** | 20 | 21 | 15 | 22 | 20 | 17 | 25 | 31 | 19 | 8 | 6 | 5 |

Values are presented as sample size (N), mean, standard deviation (SD), and 5th, 10th, 25th, 50th, and 75th percentiles. Normative scores are stratified by five age groups (50-54, 55-59, 60-64, 65-69, and 70-75 years) and three education levels (higher, apprenticeship/secondary, and primary or less).
